# Supplementary material for: Horizontal acquisition of prokaryotic hopanoid biosynthesis reorganizes membrane physiology driving lifestyle innovation in a eukaryote
Source: Nat Commun. 2025 Apr 7;16:3291. doi: 10.1038/s41467-025-58515-w (PMC11976957; doi:10.1038/s41467-025-58515-w)
Supplement: Supplementary file 1 — Supplementary Information [file 41467_2025_58515_MOESM1_ESM.pdf]

Supplementary Information

Horizontal acquisition of prokaryotic hopanoid biosynthesis reorganizes membrane physiology driving lifestyle innovation in a eukaryote

Bhagyashree Dasari Rao[1,2,#], Elisa Gomez Gil[1,2,#], Maria Peter[3], Gabor Balogh[3], Vanessa Nunes[2], James I. MacRae[2], Qu Chen[2], Peter B. Rosenthal[2] and Snezhana Oliferenko[1,2,*]

[1]Randall Centre for Cell and Molecular Biophysics, School of Basic and Medical Biosciences, King's College London, Guy's Campus, London, SE1 1UL, UK

[2]The Francis Crick Institute, 1 Midland Road, London, NW1 1AT, UK

[3] Institute of Biochemistry, HUN-REN Biological Research Centre, Temesvári krt. 62, Szeged, H-6726, Hungary

[#]These authors contributed equally

*Corresponding author: Snezhana Oliferenko

snezhana.oliferenko@kcl.ac.uk

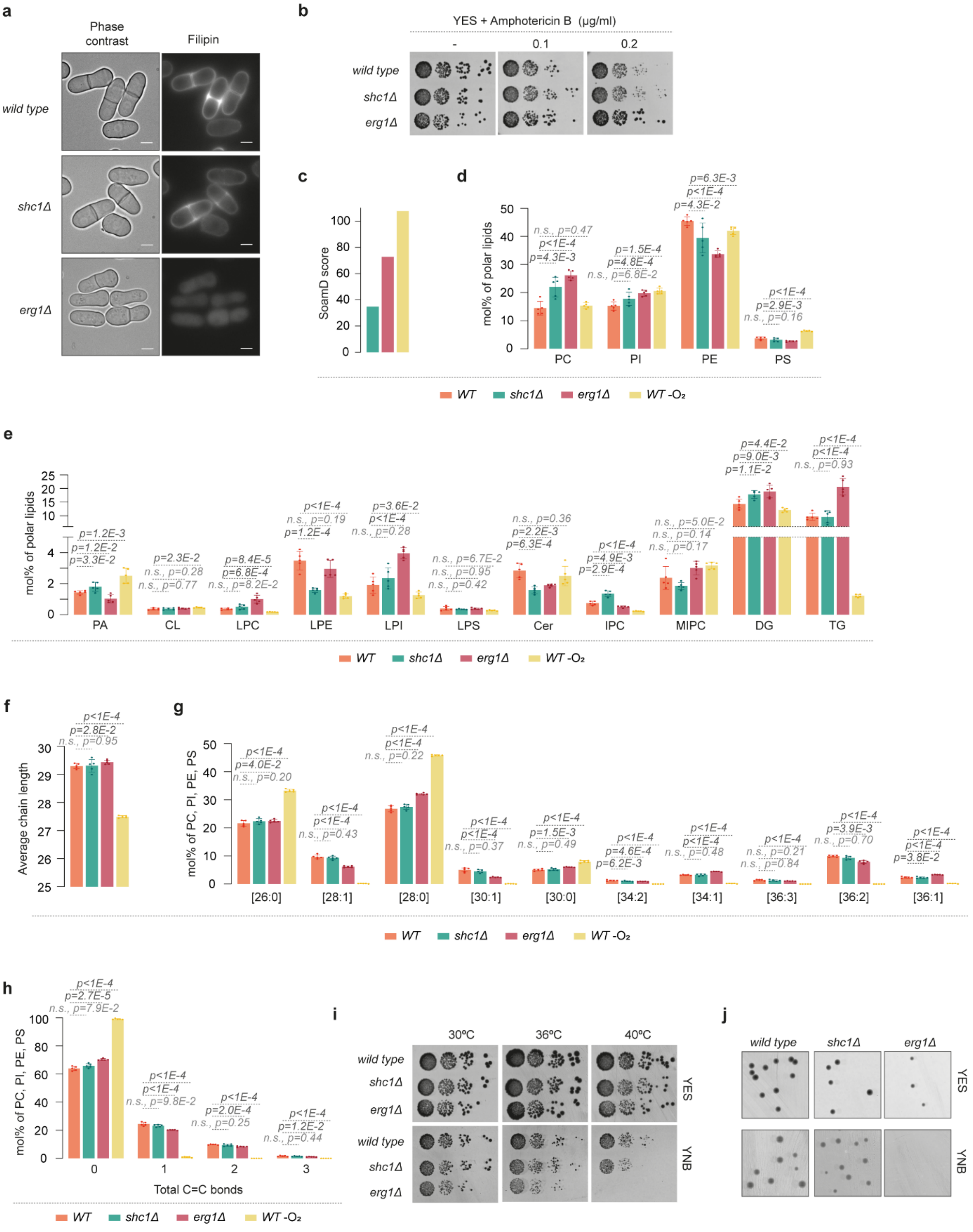

Supplementary Figure 1. *S. japonicus* lipidome responds to the lack of either ergosterol or hopanoids.

(**a**) Micrographs of filipin-stained *S. japonicus* wild type (*WT*), *shc1Δ* and *erg1Δ* cells grown in YES medium. Brightfield images are included. Scale bars represent 5 µm. (**b**) Serial dilution assay of *S. japonicus* strains of indicated genotypes carried out in YES medium in the absence or the presence of amphotericin B. (**c**) Sum of absolute

mol% difference (SoamD) relative to *S. japonicus WT* for every lipid species. (**d**) Relative abundance of the four main GPL classes (PC, PI, PE and PS) in *S. japonicus WT*, *shc1Δ* and *erg1Δ* cells grown in normoxia, and *WT* cells grown in anoxia. (**e**) Relative abundance of the indicated lipid classes: phosphatidic acid (PA), cardiolipin (CL), lysophosphatidylcholine (LPC), lysophosphatidylethanolamine (LPE), lysophosphatidylinositol (LPI), lysophosphatidylserine (LPS), ceramide (Cer), inositol phosphoceramide (IPC), mannosyl-inositolphosphoceramide (MIPC), diacylglycerol (DG) and triacylglycerol (TG) in *S. japonicus WT*, *shc1Δ* and *erg1Δ* cells grown in normoxia, and *WT* cells grown in anoxia. (**f**) Average combined FA length calculated for the sum of PC, PI, PE, and PS in *S. japonicus* strains of indicated genotypes and conditions. (**g**) Molecular species composition calculated for the sum of PC, PI, PE, and PS in *S. japonicus WT*, *shc1Δ* and *erg1Δ* cells grown in normoxia, and *WT* cells grown in anoxia. The categories are shown as the total number of carbon atoms: total number of double bonds in acyl chains. (**h**) Grouping of GPL species according to the number of double bonds calculated for the sum of PC, PI, PE, and PS in *S. japonicus WT*, *shc1Δ* and *erg1Δ* cells grown in normoxia, and *WT* cells grown in anoxia. (**i**) Serial dilution assay of *S. japonicus* strains of indicated genotypes performed in indicated conditions. (**j**) Representative images of colonies from the CFU assay shown in Fig. 1m, n. (**d**-**h**) Data are represented as average ± S.D (n=3 biological and 2 technical repeats). p-values are derived from two-tailed unpaired t-test. (**a**, **b**, **i**, **j**) Experiments were repeated three times with comparable results. Source data are provided as a Source Data file.

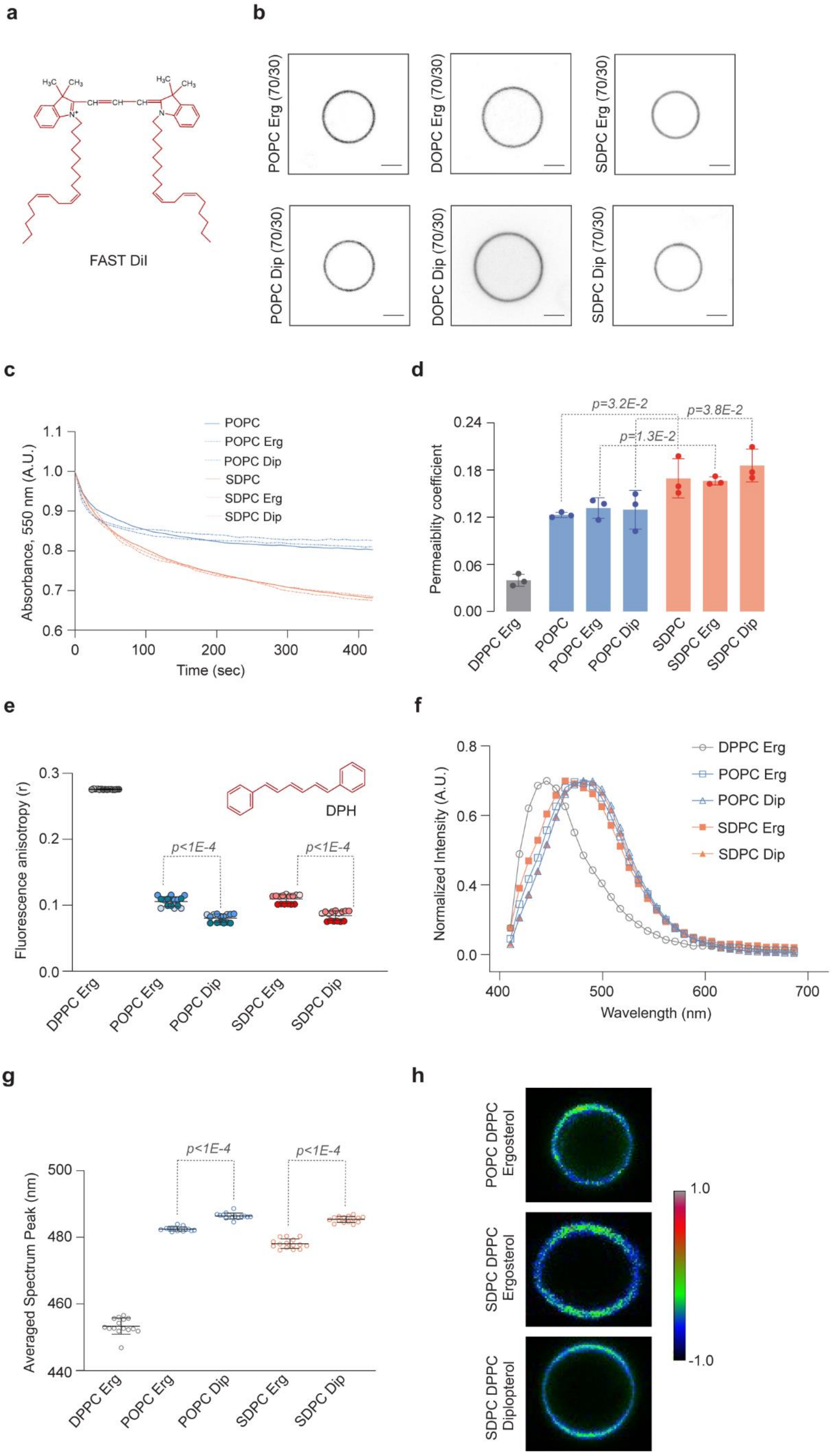
a
FAST DiI
b
POPC Erg (70/30)
DOPC Erg (70/30)
SDPC Erg (70/30)
POPC Dip (70/30)
DOPC Dip (70/30)
SDPC Dip (70/30)
c
POPC
POPC Erg
POPC Dip
SDPC
SDPC Erg
SDPC Dip
Absorbance, 550 nm (A.U.)
Time (sec)
d
p=3.2E-2
p=3.8E-2
p=1.3E-2
Permeaiblity coefficient
DPPC Erg
POPC
POPC Erg
POPC Dip
SDPC
SDPC Erg
SDPC Dip
e
Fluorescence anisotropy (r)
DPH
p<1E-4
p<1E-4
DPPC Erg
POPC Erg
POPC Dip
SDPC Erg
SDPC Dip
f
Normalized Intensity (A.U.)
Wavelength (nm)
DPPC Erg
POPC Erg
POPC Dip
SDPC Erg
SDPC Dip
g
Averaged Spectrum Peak (nm)
p<1E-4
p<1E-4
DPPC Erg
POPC Erg
POPC Dip
SDPC Erg
SDPC Dip
h
POPC DPPC Ergosterol
SDPC DPPC Ergosterol
SDPC DPPC Diplopterol
1.0
-1.0

Supplementary Figure 2. Biophysical properties of model membranes containing asymmetrical or symmetrical glycerophospholipids.

(**a**) Chemical structure of the probe 1,1'-Dilinoleyl-3,3,3',3'-Tetramethylindocarbocyanine, 4-Chlorobenzenesulfonate (FAST DiI) used for labelling GUVs in phase-separation experiments. (**b**) Representative spinning disk confocal images of two-component liposomes. Incorporation of FAST DiI into membranes assembled from either unsaturated symmetrical lipids (POPC or DOPC) or the saturated asymmetrical lipid (SDPC) with either 30 mol% ergosterol (top panel) or diplopterol (bottom panel), n=20 GUVs, one biological repeat. Mid-planes from confocal z-stacks are shown, scale bars represent 2 µm. (**c**) The representative curves for time-dependent reduction in absorbance are shown in single and two-component multilamellar vesicles made with POPC or SDPC with either 30 mol% ergosterol or diplopterol. (**d**) Permeability coefficients estimated from (**c**) (average ± S.D. from n=3 biological repeats). (**e**) Membrane order measured by DPH fluorescence anisotropy (structure shown in top right corner) in two-component LUVs made with POPC or SDPC and either ergosterol or diplopterol (average ± S.D. from n=3 biological repeats estimated using Equation (1)). DPPC-ergosterol was used as a control. (**f**) Representative normalized intensity vs wavelength curves obtained from C-laurdan imaging in two-component GUVs made with POPC or SDPC. Data for gel-like membranes made with DPPC and ergosterol is shown for comparison. (**g**) Estimation of membrane polarity from spectral GP imaging. Average spectrum peaks shown for two-component GUVs made with POPC or SDPC. Peak values were estimated from the normalized intensity vs wavelength plots (Supplementary Fig. 2e) of individual measurements (average ± S.D., n=15 GUVs, one biological repeat). (**h**) Representative pseudocoloured scanning confocal images of three-component phase separated GUVs stained with C-laurdan. Quantifications are shown in Fig. 2e-g. Lipid mixtures are indicated. Color bar indicates the range of GP values where blues show low membrane order and reds show high membrane order. (**d**, **e**, **g**) p-values were obtained by two-tailed unpaired parametric t-test. Source data are provided as a Source Data file.

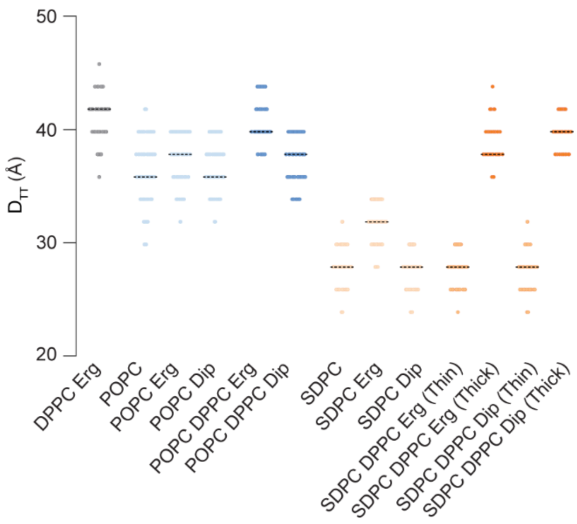

Supplementary Figure 3. Estimation of D_{TT} from cryo-EM measurements in liposomes.

Distribution of D_{TT} values from individual measurements in single, two- and three-component LUVs made with either symmetrical unsaturated POPC or asymmetrical saturated SDPC. Specific LUV compositions are indicated. Source data are provided as a Source Data file.

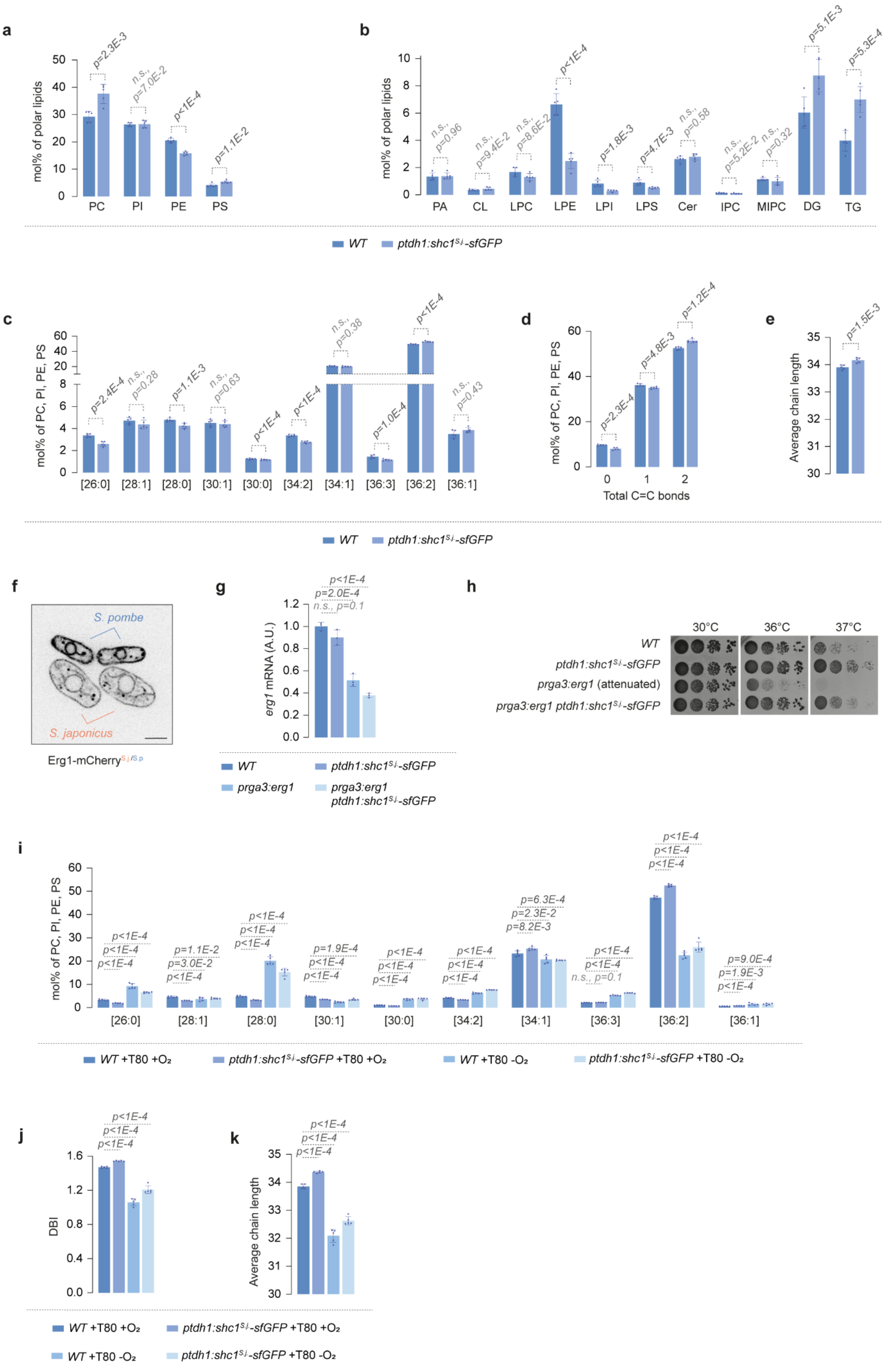

a
mol% of polar lipids
PC
PI
PE
PS
p=2.3E-3
n.s., p=7.0E-2
p<1E-4
p=1.1E-2
b
mol% of polar lipids
PA
CL
LPC
LPE
LPI
LPS
Cer
IPC
MIPC
DG
TG
n.s., p=0.96
n.s., p=9.4E-2
n.s., p=8.6E-2
p<1E-4
p=1.8E-3
p=4.7E-3
n.s., p=0.58
n.s., p=5.2E-2
n.s., p=0.32
p=5.1E-3
p=5.3E-4
WT
ptdh1:shc1S.j-sfGFP
c
mol% of PC, PI, PE, PS
[26:0]
[28:1]
[28:0]
[30:1]
[30:0]
[34:2]
[34:1]
[36:3]
[36:2]
[36:1]
p=2.4E-4
n.s., p=0.28
p=1.1E-3
n.s., p=0.63
p<1E-4
p<1E-4
n.s., p=0.38
p=1.0E-4
p<1E-4
n.s., p=0.43
d
mol% of PC, PI, PE, PS
Total C=C bonds
p=2.3E-4
p=4.8E-3
p=1.2E-4
e
Average chain length
p=1.5E-3
WT
ptdh1:shc1S.j-sfGFP
f
S. pombe
S. japonicus
Erg1-mCherryS.j/S.p
g
erg1 mRNA (A.U.)
p<1E-4
p=2.0E-4
n.s., p=0.1
WT
ptdh1:shc1S.j-sfGFP
prga3:erg1
prga3:erg1 ptdh1:shc1S.j-sfGFP
h
30°C
36°C
37°C
WT
ptdh1:shc1S.j-sfGFP
prga3:erg1 (attenuated)
prga3:erg1 ptdh1:shc1S.j-sfGFP
i
mol% of PC, PI, PE, PS
[26:0]
[28:1]
[28:0]
[30:1]
[30:0]
[34:2]
[34:1]
[36:3]
[36:2]
[36:1]
p<1E-4
p=1.1E-2
p=3.0E-2
p=1.9E-4
p=6.3E-4
p=2.3E-2
p=8.2E-3
n.s., p=0.1
p=9.0E-4
p=1.9E-3
WT +T80 +O2
ptdh1:shc1S.j-sfGFP +T80 +O2
WT +T80 -O2
ptdh1:shc1S.j-sfGFP +T80 -O2
j
DBI
p<1E-4
k
Average chain length
p<1E-4
WT +T80 +O2
ptdh1:shc1S.j-sfGFP +T80 +O2
WT +T80 -O2
ptdh1:shc1S.j-sfGFP +T80 -O2

Supplementary Figure 4. *S. pombe* lipidome adapts to the presence of hopanoids.

(**a**) Relative abundance of the four main GPL classes (PC, PI, PE and PS) in *S. pombe* cells of indicated genotypes. (**b**) Relative abundance of the indicated lipid classes (PA, CL, LPC, LPE, LPI, LPS, Cer, IPC, MIPC, DG and TG) in *S. pombe* wild type (*WT*) and *ptdh1:shc1*$^{S.j.}$*-sfGFP* cells. (**c**) Molecular species composition for the sum of PC, PI, PE, and PS in *S. pombe WT* and *ptdh1:shc1*$^{S.j.}$*-sfGFP* cells, shown as total number of carbon atoms: total number of double bonds in acyl chains. (**d**) Grouping of GPL species according to the number of double bonds calculated for the sum of PC, PI, PE, and PS in *S. pombe WT* and *ptdh1:shc1*$^{S.j.}$*-sfGFP* cells. (**e**) Average combined FA length calculated for the sum of PC, PI, PE, and PS. (**f**) Single plane spinning disk confocal image of *S. japonicus* and *S. pombe* expressing Erg1-mCherry grown in YES. Scale bars represent 5 µm. (**g**) Steady-state *erg1* mRNA levels in *S. pombe* strains of indicated genotypes grown in YES, as measured by qPCR, normalized to the *WT*. Results are shown as average ± S.D. (n=3 biological repeats). (**h**) Serial dilution assay of *S. pombe* strains of indicated genotypes carried out at indicated temperatures in YES medium. (**i**) Molecular species composition calculated for the sum of PC, PI, PE, and PS in *S. pombe WT* and *ptdh1:shc1*$^{S.j.}$*-sfGFP* strains grown in normoxia or in anoxia in the presence of Tween 80. The categories are shown as the total number of carbon atoms: total number of double bonds in acyl chains. (**j**) Comparison of the double bond indexes (DBI) calculated for the four main GPL classes in *S. pombe* strains of the indicated genotypes and conditions. (**k**) Average combined FA length calculated for the sum of PC, PI, PE, and PS in *S. pombe* strains of the indicated genotypes and conditions. (**a**-**e, i-k**) Data are represented as average ± S.D. (n=3 biological and 2 technical repeats). (**a**-**e, g, i-k**) p-values are derived from two-tailed unpaired t-test. (**f**, **h**). Experiments were repeated three times with similar results. Source data are provided as a Source Data file.
