# Supplementary material for: Horizontal acquisition of prokaryotic hopanoid biosynthesis reorganizes membrane physiology driving lifestyle innovation in a eukaryote
Source: Nat Commun. 2025 Apr 7;16:3291. doi: 10.1038/s41467-025-58515-w (PMC11976957; doi:10.1038/s41467-025-58515-w)
Supplement: Supplementary file 2 — Description of Additional Supplementary Files [file 41467_2025_58515_MOESM2_ESM.pdf]

Description of Additional Supplementary Files

File name: Supplementary Data 1
Description: Lipidomics. All data presented as Excel tables. (**a**) Detailed lipidomics dataset for comparison of *S. japonicus wild type* (*WT*), *shc1Δ* and *erg1Δ* cells and *WT* cells in the absence of oxygen. (**b**) Detailed lipidomics dataset for comparison of *S. pombe WT* and *ptdh1:shc1*$^{S.j.}$*-sfGFP* cells. Data are expressed as mol% of polar lipids; polar lipids include all measured lipids except DG, TG, EE, and sterols. Sterols were analyzed by GC-MS, all other lipids by shotgun ESI-MS. Data are expressed as average ± S.D., n= 5 (three biological and two technical replicates). Student's t-tests were performed for pairwise multiple comparisons; significance was accepted for $p < 0.05$. Blue fill indicates $p < 0.05$. PC, phosphatidylcholine; PE, phosphatidylethanolamine; MMPE, monomethyl PE; DMPE, dimethyl-PE; PI, phosphatidylinositol; PS, phosphatidylserine; PG, phosphatidylglycerol; PA, phosphatidic acid; CL, cardiolipin; LPC, LPE, LPI, LPS, LPA, LCL, the corresponding lyso lipids; Cer, ceramide; IPC, inositolphosphoceramide; MIPC, mannosyl-inositolphosphoceramide; DG, diacylglycerol; TG, triacylglycerol; EE, ergosteryl ester. DBI, double bond index; Av_ChL, average chain length. Sum formulas for glycero(phospho)lipids (GPL) are defined as the lipid class abbreviation followed by the total number of carbons and total number of double bonds for all chains, e.g. PC(34:1). Sum formulas for sphingolipids are defined as the lipid class abbreviation followed by the total number of carbons, total number of double bonds and total number of hydroxyl groups in the long chain base and the fatty acyl moiety, e.g. Cer(44:0:3). Double bond index (DBI) was calculated for GPLs (PC, PE, PI and PS) as $\Sigma(\text{db} \times [\text{GPLi}])/\Sigma[\text{GPLi}]$, where db is the total number of double bonds in fatty acyls in a given GPL species, and the square bracket indicates mol% of GPLs. Average acyl chain length (Av_ChL) was calculated for GPLs (PC, PE, PI and PS) as $\Sigma(\text{C} \times [\text{GPLi}])/\Sigma[\text{GPLi}]$, where C is the total number of carbons in fatty acyls in a given GPL, and the square bracket indicates mol% of GPLs. Average species profile was calculated for PC, PE, PI and PS. SoamD score was calculated for all lipid species as $\Sigma\text{abs}(\text{mol\%}_{\text{mutant}} - \text{mol\%}_{WT})$. Please note that the list of lipid species is matched for the two yeast species. Due to the substantial difference in their lipid profile, one can find zero values for certain lipid species in *S. japonicus* or *S. pombe*. (**c**) Detailed triterpenoid abundance dataset for comparison of *S. pombe WT*, *ptdh1:shc1*$^{S.j.}$*-sfGFP*, *prga3:erg1* and *ptdh1:shc1*$^{S.j.}$*-sfGFP prga3:erg1* cells analyzed by GC-MS. Data are expressed µM/OD, average ± S.D., n= 3 (two biological and one technical replicates). (**d**) Detailed lipidomics dataset for comparison of *S. pombe WT* and *ptdh1:shc1*$^{S.j.}$*-sfGFP* cells supplemented with Tween 80, grown in normoxia or upon shift to anoxia. Data are expressed as mol% of polar lipids; polar lipids include all measured lipids except DG, TG, EE, and sterols. Sterols were analyzed by GC-MS, all other lipids by shotgun ESI-MS. Data are expressed as average ± S.D., n= 5 (three biological and two technical replicates). Student's t-tests were performed for pairwise multiple comparisons; significance was accepted for $p < 0.05$. Blue fill indicates $p < 0.05$. DBI, double bond index; Av_ChL, average chain length. Sum formulas for glycero(phospho)lipids (GPL) are defined as the lipid class abbreviation followed by the total number of carbons and total number of double bonds for all chains, e.g. PC(34:1). Sum formulas for sphingolipids are defined as the lipid class abbreviation followed by the total number of carbons, total number of double bonds and total number of hydroxyl groups in the long chain base and the fatty acyl moiety, e.g.

Cer(44:0:3). Double bond index (DBI) was calculated for GPLs (PC, PE, PI and PS) as Σ(db x [GPLi])/Σ[GPLi], where db is the total number of double bonds in fatty acyls in a given GPL species, and the square bracket indicates mol% of GPLs. Average acyl chain length (Av_ChL) was calculated for GPLs (PC, PE, PI and PS) as Σ(C x [GPLi])/Σ[GPLi], where C is the total number of carbons in fatty acyls in a given GPL, and the square bracket indicates mol% of GPLs. Average species profile was calculated for PC, PE, PI and PS. SoamD score was calculated for all lipid species as $\Sigma abs(mol\%_{mutant} - mol\%_{WT})$. Please note that the list of lipid species is matched for the two yeast species. Due to the substantial difference in their lipid profile, one can find zero values for certain lipid species in *S. japonicus* or *S. pombe*. (**e**) GC-MS quantification details. (**f**) ESI-MS quantification details. Source data are provided as a Source Data file.

File name: Supplementary Data 2
Description: List of fission yeast strains used in this study. *S. japonicus* and *S. pombe* strains used in each Figure are listed in individual spreadsheets in order of appearance.

File name: Supplementary Data 3
Description: List of primers used in this study. Primers used to generate deletions, fluorescent protein tagging and promoter replacement, and genotyping primers are listed in individual spreadsheets.
