# Supplementary material for: Horizontal acquisition of prokaryotic hopanoid biosynthesis reorganizes membrane physiology driving lifestyle innovation in a eukaryote
Source: Nat Commun. 2025 Apr 7;16:3291. doi: 10.1038/s41467-025-58515-w (PMC11976957; doi:10.1038/s41467-025-58515-w)
Supplement: Supplementary file 6 — Reporting Summary [file 41467_2025_58515_MOESM6_ESM.pdf]

nature portfolio

Corresponding author(s): Snezhana Oliferenko

Last updated by author(s): Mar 13, 2025

Reporting Summary

Nature Portfolio wishes to improve the reproducibility of the work that we publish. This form provides structure for consistency and transparency in reporting. For further information on Nature Portfolio policies, see our Editorial Policies and the Editorial Policy Checklist.

Statistics

For all statistical analyses, confirm that the following items are present in the figure legend, table legend, main text, or Methods section.

n/a	Confirmed	
☐	☒	The exact sample size (*n*) for each experimental group/condition, given as a discrete number and unit of measurement
☐	☒	A statement on whether measurements were taken from distinct samples or whether the same sample was measured repeatedly
☐	☒	The statistical test(s) used AND whether they are one- or two-sided<br>*Only common tests should be described solely by name; describe more complex techniques in the Methods section.*
☒	☐	A description of all covariates tested
☒	☐	A description of any assumptions or corrections, such as tests of normality and adjustment for multiple comparisons
☐	☒	A full description of the statistical parameters including central tendency (e.g. means) or other basic estimates (e.g. regression coefficient) AND variation (e.g. standard deviation) or associated estimates of uncertainty (e.g. confidence intervals)
☒	☐	For null hypothesis testing, the test statistic (e.g. *F*, *t*, *r*) with confidence intervals, effect sizes, degrees of freedom and *P* value noted<br>*Give P values as exact values whenever suitable.*
☒	☐	For Bayesian analysis, information on the choice of priors and Markov chain Monte Carlo settings
☒	☐	For hierarchical and complex designs, identification of the appropriate level for tests and full reporting of outcomes
☒	☐	Estimates of effect sizes (e.g. Cohen's *d*, Pearson's *r*), indicating how they were calculated

Our web collection on statistics for biologists contains articles on many of the points above.

Software and code

Policy information about availability of computer code

Data collection	Epifluorescence images were acquired using Zen Pro 2012 (blue edition, Carl Zeiss Microscopy GmbH). Spinning-disk confocal images were acquired using Andor Fusion 2.3.0.44 (Oxford Instruments). Confocal images for measurements of membrane order in live cells were acquired using NIS Elements (Nikon). Electron microscopy grids were imaged using EPU software v 2.11 (Thermo). Western blots scans were acquired using ChemiDoc MP imaging system (Bio-Rad). GC-MS data were acquired using MassHunter Workstation software (B.06.00 SP01, Agilent Technologies). ESI-MS data were acquired using the LipidXplorer software (open source, PMID: 22272252). No custom algorithms or software were used.
Data analysis	Fiji v1.54g was used for analysis of all microscopy images, colony formation assays and Western blot scans. Cryo-EM movies were imported into Relion v 4.0.0 (PMID: 34783343), followed by Relion's own motion correction and CTF estimation (CTFFIND, v 4.1.13, PMID: 26278980), and subsequent analyses in Fiji v1.54g. Triterpenoid identification by GC-MS was performed by comparison to retention time and fragment ion pattern of authentic standards using MassHunter Workstation software (B.06.00 SP01, Agilent Technologies) and confirmed by comparison to deconvoluted mass spectra of those in the NIST Mass Spectral Library software (NIST 23, software version 3.0). ESI-MS lipidomics analyses were performed using LipidXplorer software (open source, PMID: 22272252). Growth rates and Tmid were calculated using the Growthcurver R package (PMID: 27094401). Prism10.4.1 (GraphPad) was used for all statistical analyses.No custom algorithms or software were used.

For manuscripts utilizing custom algorithms or software that are central to the research but not yet described in published literature, software must be made available to editors and reviewers. We strongly encourage code deposition in a community repository (e.g. GitHub). See the Nature Portfolio guidelines for submitting code & software for further information.

Data

Policy information about availability of data

All manuscripts must include a data availability statement. This statement should provide the following information, where applicable:

- Accession codes, unique identifiers, or web links for publicly available datasets
- A description of any restrictions on data availability
- For clinical datasets or third party data, please ensure that the statement adheres to our policy

All data presented in graphs and uncropped scans of all blots generated in this study are included in the Source Data file. Lipidomics data are provided in Supplementary Data 1 file. All microscopy, cryo-EM and Western blotting data have been deposited in the Figshare database under accession code 10.6084/m9.figshare.c.7668101. Raw lipidomics data have been deposited in the Zenodo database under accession code 10.5281/zenodo.15017552. Source data are provided with this paper.

Research involving human participants, their data, or biological material

Policy information about studies with human participants or human data. See also policy information about sex, gender (identity/presentation), and sexual orientation and race, ethnicity and racism.

Reporting on sex and gender	n/a
Reporting on race, ethnicity, or other socially relevant groupings	n/a
Population characteristics	n/a
Recruitment	n/a
Ethics oversight	n/a

Note that full information on the approval of the study protocol must also be provided in the manuscript.

Field-specific reporting

Please select the one below that is the best fit for your research. If you are not sure, read the appropriate sections before making your selection.

- [x] Life sciences
- [ ] Behavioural & social sciences
- [ ] Ecological, evolutionary & environmental sciences

For a reference copy of the document with all sections, see nature.com/documents/nr-reporting-summary-flat.pdf

Life sciences study design

All studies must disclose on these points even when the disclosure is negative.

Sample size	No sample-size calculations were performed. Sample sizes are based on our experience and community standards. As can be seen from our data, the possible diversity of cellular populations and lipid vesicles is adequately represented in our samples.
Data exclusions	No data were excluded in the cell biological, physiological and biophysical experiments. In the GC-MS and ESI-MS experiments, linear regression analyses between sample OD595 and lipid content were performed for each experimental group. Samples showing anomalous lipid levels relative to the sample amount were excluded as likely sample preparation artefacts.
Replication	At least three independent biological replicates were performed per experiment, with reproducible results. For confocal microscopy experiments with GUVs, measurements were carried out on individual GUVs from either one or two preparations. For cryo-EM measurements, individual LUVs were analyzed from a single preparation. In case of fluorescence anisotropy and permeability measurements, at least 3 or more sets of samples were used for measurements. The results were found to be reproducible in all cases.
Randomization	Samples were allocated based on genotypes of the fission yeast strains used.
Blinding	The investigators were not blinded to group allocation. Experiments were performed according to community standards. Blinding was not relevant to our study.

Reporting for specific materials, systems and methods

We require information from authors about some types of materials, experimental systems and methods used in many studies. Here, indicate whether each material, system or method listed is relevant to your study. If you are not sure if a list item applies to your research, read the appropriate section before selecting a response.

Materials & experimental systems

n/a	Involved in the study	
☐	☒	Antibodies
☒	☐	Eukaryotic cell lines
☒	☐	Palaeontology and archaeology
☐	☒	Animals and other organisms
☒	☐	Clinical data
☒	☐	Dual use research of concern
☒	☐	Plants

Methods

n/a	Involved in the study	
☒	☐	ChIP-seq
☒	☐	Flow cytometry
☒	☐	MRI-based neuroimaging

Antibodies

Antibodies used

Mouse α-RFP (Chromotek; catalogue no: 6G6-20, clone ID: 6G6). Validation and relevant citations are available at the manufacturer's website (https://www.ptglab.com/products/RFP-antibody-6G6.htm).

IRDye 800CW goat α-mouse IgG secondary antibody (Li-Cor; catalogue no: 926-32210).

Validation

Validation and relevant citations are available at the manufacturer's website (https://www.ptglab.com/products/RFP-antibody-6G6.htm). Primary anti-RFP antibody was also validated by Western blotting using non-tagged mCherry strains alongside the mCherry-tagged strain for each species.

Animals and other research organisms

Policy information about studies involving animals; ARRIVE guidelines recommended for reporting animal research, and Sex and Gender in Research

Laboratory animals

The study did not involve laboratory animals.

Wild animals

The study did not involve wild animals.

Reporting on sex

n/a

Field-collected samples

The study did not involve samples collected from the field.

Ethics oversight

No ethical approval or guidance were required. The research organisms are two species of fission yeast.

Note that full information on the approval of the study protocol must also be provided in the manuscript.

Plants

Seed stocks

n/a

Novel plant genotypes

n/a

Authentication

n/a
